# Prevalence and Associated Factors of Suicidal Ideation and Attempt among People Living with HIV/AIDS at Zewditu Memorial Hospital, Addis Ababa, Ethiopia: A Cross-Sectional Study

**DOI:** 10.1155/2017/2301524

**Published:** 2017-04-11

**Authors:** Etsay Hailu Gebremariam, Mebratu Mitiku Reta, Zebiba Nasir, Fisseha Zewdu Amdie

**Affiliations:** ^1^Department of Psychiatry, School of Medicine, University of Gondar, Gondar, Ethiopia; ^2^Amanuel Specialized Mental Health Hospital, Addis Ababa, Ethiopia; ^3^Department of Internal Medicine, University of Gondar, Gondar, Ethiopia; ^4^Department of Nursing, School of Nursing, University of Gondar, Gondar, Ethiopia

## Abstract

*Background*. Human Immune Deficiency Virus (HIV/AIDS) continues to be an underrecognized risk for suicidal ideation, suicidal attempt, and completion of suicide. Suicidal ideation and attempt in HIV/AIDS is not only a predictor of future attempted suicide and completed suicide.* Methods*. An institution based cross-sectional study was conducted among HIV-positive patients attending HIV care at Zewditu Memorial Hospital. Systematic random sampling technique was used to recruit 423 participants from April to May 2014. Composite International Diagnostic Interview was used to collect data. Multivariable logistic regression was computed to assess factors associated with suicidal ideation and attempt.* Result*. Suicidal ideation and suicidal attempt were found to be 22.5% and 13.9%, respectively. WHO clinical stage of HIV, not being on HAART, depression, family history of suicidal attempt, and perceived stigma were associated with suicidal ideation. WHO clinical stage, being female, not being on HAART, use of substance, and depression were associated with suicidal attempt.* Conclusion*. Early diagnosis and treatment of opportunistic infections, depression, and early initiation of ART need to be encouraged in HIV-positive adults. Furthermore, counseling on substance use and its consequences and early identification of HIV-positive people with family history of suicidal ideation have to be considered.

## 1. Background

Globally, every year around 800,000 to a million people die due to suicide [1]. Suicide is the 10th leading cause of death worldwide and third leading cause of death among those aged 15–44 years. It represented 1.8% of global burden of disease in 1998 [1–3]. It is also predicted that, by 2020, the rate of death due to suicide will be increased to one every 20 seconds [4]. Currently, more than 36.9 million people live with HIV/AIDS worldwide [5, 6] and around 25.5 million (69%) HIV-positive people are living in Sub-Saharan Africa [7, 8]. In Ethiopia, the prevalence of HIV among adult population was 1.2% (1.6% for females and 0.8% for males) in 2014 [9]. People living with HIV/AIDS are high-risk groups of suicidal ideation and attempt. Suicidal ideation is an important phase in the suicidal process prior to completed suicide. Compared to the general population, people living with HIV/AIDS have 7 to 36 times greater risk of completed suicide. Suicidal ideation in HIV/AIDS is a predictor of future suicidal attempt and completed suicide and it is associated with reduced quality of life and poor adherence to antiretroviral therapy [10, 11]. It has a long-standing consequence for psychological trauma in children, friends, and relatives. Suicidality also determines economic productivity [12]. HIV/AIDS continues to be an underrecognized risk for suicidal ideation, suicidal attempt, and completed suicide [13]. The prevalence of suicide among HIV-positive population is still high despite good prognosis of their quality of life through the introduction of ART [14]. In Ethiopia, the prevalence and associated factors of suicidal ideation and attempt among HIV-positive people are not well known. Therefore, assessing the magnitude of suicidal ideation and suicidal attempt and identifying factors associated with them may have paramount benefit in preventing their further social and economic consequences. Data from this study provides important information which helps to design appropriate intervention.

## 2. Methods

### 2.1. Study Set-Up

An institution based cross-sectional study was conducted at Zewditu Memorial Hospital. Zewditu Memorial Hospital is one of the five Federal Hospitals located in Cherkos Kifle Ketema at the center of Addis Ababa, capital city of Ethiopia. It is the first hospital where ART was stated for the first time in Ethiopia since 2005. There were 14,347 HIV-positive patients having HIV care follow-up in the hospital in 2014. Participants were eligible to participate in the survey if they were aged 18 years or above and had a follow-up visit at Zewditu hospital during the study period.

### 2.2. Sampling Procedure

The sample size was determined by using single population proportion. The calculated sample size was 423. A systematic random sampling technique was used to select study participants. Every twelve clients were interviewed during the period from May to June 2014. Interviewer-administered questionnaire was used to collect data. Participants' chart was also reviewed to record their date of HIV diagnosis, WHO clinical stage, and CD4 count of the patient.

### 2.3. Data Collection Tools and Procedures

Composite International Diagnostic Interview (CIDI) adopted by World Mental Health (WMH) Survey Initiative version of the World Health Organization (WHO) which was evaluated in Ethiopia in 2002 was used to assess suicidal ideation and attempt among HIV-positive patients. Suicidal ideation was recorded if the respondents respond with "yes" to the following question: have you ever seriously thought about committing suicide? And suicidal attempt was recorded if the respondents respond with "yes" to the following question: have you ever attempted suicide? Patient Health Questionnaire (PHQ-9) was used to assess depression in study participants. PHQ-9 is a toll which contains 9 questions. Each question has values from 0 to 4: 0 means not at all, 1 means several days, 2 means more than half the days, and 4 means nearly every day. The scores in each of the columns were added together. The scores of each column in the PHQ-9 Questionnaire were added together and those who scored 5 to 27 were considered to have depression. Perceived stigma was screened with HIV-related stigma scale. WHO clinical stages were defined as WHO clinical stage I = asymptomatic (no significant immune suppression), WHO clinical stage II = mild clinical symptoms (mild immune suppression), WHO clinical stage III = advanced immune suppression, and WHO clinical stage IV = severe immune suppression with severe clinical symptoms. Primarily the questionnaire was translated from English to Amharic and then back to English by another language instructor to maintain its consistency. Finally, Amharic version of the questionnaire was used to interview patients.

### 2.4. Data Analysis

The filled questionnaires were checked manually for completeness, coded and entered into EPI Info version 7 statistical software, and exported to SPSS version 20 for further analysis. Descriptive statistics were computed to explain the study population with respect to the relevant variables. Variables having a *p* value < 0.2 in bivariate analysis were fitted to multivariable logistic regression. Odds ratio with their 95% CI was computed. Statistical significance was inferred at *P* value < 0.05.

### 2.5. Ethical Approval

Ethical clearance was obtained from an institutional review board of University of Gondar and Amanuel Specialized Mental Health Hospital. Official letter was written to Zewditu Memorial Hospital. Privacy and confidentiality of the study participants were maintained. Participants were fully informed about the aims of the study before the start of the interview and verbal informed consent was obtained from them.

## 3. Results

### 3.1. Sociodemographic Characteristics of the Study Participants

A total of 417 participants were involved in the study with a response rate of 98.6%. The mean age of respondents was found to be 40.5 (SD: ±8.86) years. Two hundred forty-three (58.3%) of the respondents were female. Two hundred three (48.7%) of the participants were unemployed. Three hundred forty-two (82.0%) of the participants knew their HIV status for at least 36 months prior to the interview. Three hundred twelve (74.8%) of respondents had a CD4 count greater than or equal to 350, and 181 (43.4%) of them were of WHO clinical stage I. Three hundred seventy-nine (90.9%) patients were on HAART.

Thirty-six (8.6%) and 5 (1.2%) of the participants had family history of suicidal attempt and suicidal commitment in the past, respectively. One hundred ninety-seven (47.2%) of the respondents had depression and 234 (56.1%) had perceived stigma due to their HIV status. Sixty-eight (16.3%) of the respondents had a history of substance use at least once in their lifetime. Fifty-two (12.5%) of the respondents were chewing khat and 27 (6.5%) of them had a history of alcohol. Only seven (1.7%) participants were current users of substances for a nonmedical purpose (Table 1).

### 3.2. Prevalence of Suicidal Ideation and Suicidal Attempt

**Figure 1:**
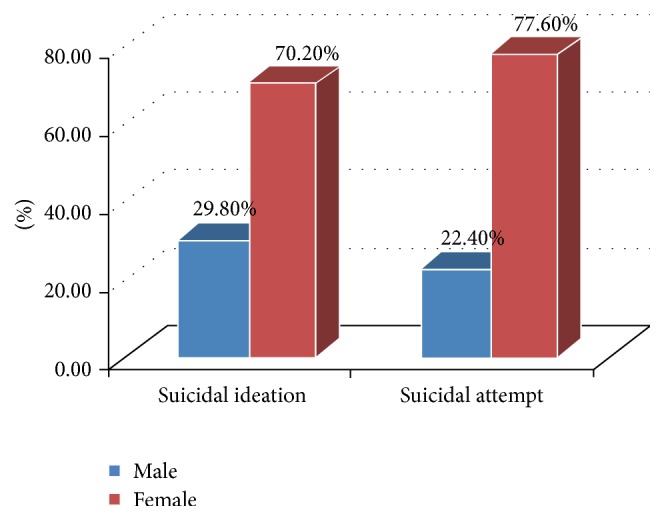
Gender proportion of suicidal ideation and attempt among HIV-positive patients at Zewditu Memorial Hospital, Addis Ababa, Ethiopia, June 2014.

Ninety-four (22.5%) and 58 (13.9%) of the study participants had suicidal ideation and suicidal attempt, respectively. Twenty-two (23.4%) of the respondents having suicidal ideation reported that they had it within 3 months after they knew their serostatus. Among the respondents who had suicidal ideation, 66 (70.2%) of them were female. Meanwhile, 13 (22.4%) respondents attempted suicide within 3 months after they knew their positive HIV test result. Among the respondents who attempted suicide, 30 (51.7%) of them had a plan to commit suicide. Among those who attempted suicide, 45 (77.6%) were female (Figure 1).

### 3.3. Factors Associated with Suicidal Ideation

This study showed that the more the progression of the HIV infection the higher the suicidal ideation and attempt. Those HIV-positive patients with WHO clinical stage IV condition were 6.5 times more likely to have suicidal ideation as compared to those patients who were asymptomatic (WHO clinical stage I) (AOR = 6.55, 95% CI: 2.35–18.20). Furthermore, those patients with WHO clinical stage III condition were 4 times more likely to have suicidal ideation as compared to those who were asymptomatic (WHO clinical stage I) (AOR = 4.12, 95% CI: 2.07–8.16). HIV-positive patients who were not on HAART were 2.5 times more likely to have suicidal ideation as compared to those who were on HAART (AOR = 2.49, 95% CI: 1.07–5.70). HIV-positive patients who had past family history of suicidal attempt were about 2.3 times more likely to have suicidal ideation as compared to those who had no past family history of suicidal attempt (AOR = 2.25, 95% CI: 1.01–5.03). Patients having comorbid depression were 2.5 times more likely to have suicidal ideation as compared to patients with no depression (AOR = 2.45, 95% CI: 1.45–4.12). Moreover, patients who had perceived stigma were 1.8 times more likely to have suicidal ideation as compared to patients who did not have perceived stigma (AOR = 1.76, 95% CI: 1.02–3.03) (Table 2).

### 3.4. Factors Associated with Suicidal Attempt

In a multivariable analysis, HIV-positive patients with WHO clinical stage IV condition were 11 times more likely to have suicidal attempt as compared to those who were asymptomatic (WHO clinical stage I) (AOR = 10.98, 95% CI: 3.56–33.79). This study also found that patients who had WHO clinical stage III condition were 4.5 times more likely to attempt suicide as compared to those who were asymptomatic (WHO clinical stage I) (AOR = 4.46, 95% CI: 1.93–10.29). HIV-positive females were 4.5 times more likely to attempt suicide (AOR = 4.48, 95% CI: 1.85–10.81). Patients who were not on HAART were 3.4 times more likely to attempt suicide (AOR = 3.44, 95% CI: 1.33–8.89). Participants who used substance at least once in their life were 3.39 times more likely to attempt suicide as compared to those who never used any substance in their lifetime (AOR = 3.39, 95% CI: 1.32–8.73). Patients having comorbid depression were 2 times more likely to attempt suicide as compared to patients who had no depression (AOR = 2.04, 95% CI: 1.07–3.87) (Table 3).

## 4. Discussion

In this study, the prevalence of suicidal ideation among HIV-positive individuals was found to be 22.5%. It was higher than that in a community-based study conducted in Addis Ababa among the adult general population (2.7%) in 1994 [15]. This variation may be due to a difference in the study participant's health status. It is also higher than the study conducted among HIV-positive individuals at Birmingham and Washington (6.5%) [13]. This might be due to sociocultural and economical difference of the study population and different tools used to screen suicidal ideation and people in Birmingham and Washington may have better HIV care including early HAART access.

The prevalence of suicidal ideation at Zewditu Memorial Hospital was lower than that in a study conducted in Nigeria, 34.7% [16], UK (southeast London), 31% [17], South Korea, 44% [18], and Texas, 59% [19]. These differences could be attributed to the variation in culture. In those countries, participants may report their suicidality experience openly and there is a difference in sample size and study design.

This finding was almost in line with the studies conducted in South Africa among HIV-positive patients at HIV counseling and testing clinic, 17.1% [20], Chelsea among HIV-positive population, 26.9% [21], North America, 26% [22], Duke University, the University of Alabama at Birmingham (UAB), Northern Outreach Clinic (NOC) in Henderson, North Carolina, and the University of North Carolina at Chapel Hill (UNC), 23% [23], as well as HIV-positive individuals in four US cities (San Francisco, Los Angeles, Milwaukee, and New York City), 19% [24]. However, it is higher than that in a study conducted in Mbarara, Uganda, among HIV-positive patients, 10% [25]. This variation may be due to variation in age of the participants.

Fifty-eight (13.9%) of the respondents had suicidal attempt. This is almost similar to the study done in South Korea, 11% [18]. However, it is higher than community-based studies conducted in Addis Ababa, 0.9% [13], and in Butajira (southern Ethiopia), 3.2% [26]. This discrepancy might be due to a difference in study groups, since our study focused on HIV-positive people. Also, our study demonstrated higher rate of suicidal attempt compared to study conducted in Uganda, which was 3.9% [11]. This variation might be due to a different tool they used and sociocultural differences.

The study also revealed that suicidal attempt among HIV-positive patients was in line with other studies done in Nigeria, 9.3% [15], whereas it was lower than a study conducted in Chelsea, UK, 20% [21]. This variation might be due to methods used in the study, cultural variation related to participants' disclosure experience to suicidal attempt, difference in study period, and sample size of the study. Our finding is also much lower than other studies conducted in Texas, 29.6% [19], and New York, 26% [27]. This variation may be due to the difference in study population and method they used.

WHO clinical stage of HIV was significantly associated with suicidal ideation. Those who had WHO clinical stage IV condition were 6.5 times more likely to have suicidal ideation as compared to those who were asymptomatic (WHO clinical stage I) (AOR = 6.55, 95% CI: 2.35–18.20) and those who had WHO clinical stage III condition were 4 times more likely to have suicidal ideation as compared to those who were asymptomatic (WHO clinical stage I) (AOR = 4.12, 95% CI: 2.07–8.16). This may be due to the fact that clinical stages are classified based on the presence and absence of opportunistic infections. HIV-positive patients who are on advanced clinical disease may have decreased quality of life which may lead them to think of death. This is supported by other similar studies conducted in Benin City, Nigeria. This finding is also in line with the study conducted in New York among HIV-positive women and those who were not on HAART [15, 27–29].

Not being on HAART was significantly associated with suicidal ideation. HIV-positive patients who were not on HAART were 2.5 times more likely to have suicidal ideation as compared to those who were on HAART (AOR = 2.49, 95% CI: 1.07–5.70). That is to say, patients may assume the future burden of HAART in terms of long-term side effects and pill burden. So they may have suicidal ideation. Another possible reason may be that those who are not on HAART may have a thought of burden of lifelong treatment in addition to HIV infection itself. Those HIV-positive patients with comorbid depression were 2.5 times more likely to have suicidal ideation as compared to those with no depression and this is supported by the study conducted in four US cities [24]. This may be due to the fact that depression aggravates the suicidality of HIV-positive patients.

This study also showed that those who had a family history of suicidal attempt were two times more likely to have suicidal ideation as compared to those with no family history of suicidal attempt (AOR = 2.25, 95% CI: 1.01–5.03). This may be due to the fact that genetic predisposing increases the risk for suicidal ideation, which is supported by international scholars [3].

Comorbid depression was found to be significantly associated with suicidal ideation. HIV-positive patients who had depression were about 2.5 times more likely to have suicidal ideation as compared to those who had no depression (AOR = 2.45, 95% CI: 1.45–4.12). This is supported by other studies done in New York and Virginia University [11, 30]. This could also be due to the fact that depressed individuals have neurotransmitter disturbance in the brain which might be contributed to hopelessness, guilt, and worthlessness which may again expose them to suicidal ideation [3].

HIV-positive patients who perceived stigma were two times more likely to have suicidal ideation as compared to those who did not (AOR = 1.76, 95% CI: 1.02–3.03), which is in line with studies conducted in Sub-Saharan Africa, Virginia University, and South Africa [7, 31]. This may be due to the fact that HIV-positive patients feel stigmatized and this may contribute to frequent psychological stress, which finally leads to suicidal ideation.

Being female, WHO clinical stage, not being on HAART, depression, and ever uses of substance were significantly associated with suicidal attempt among HIV-positive patients.

Females were 4.5 times more likely to attempt suicide as compared to males (AOR = 4.48, 95% CI: 1.85–10.81). This disagrees with other studies conducted in South Africa and Sub-Saharan Africa [4]. This may be due to the burden of household responsibilities in females and females are dependent on males, so these may contribute to having suicidal attempt. In addition, male to female proportion of the study participants may overestimate suicidal attempt in females.

WHO clinical stage was significantly associated with a suicidal attempt. Respondents who had WHO clinical stage IV condition and WHO clinical stage III condition were 11 and 4.5 times more likely to attempt suicide as compared to those who were asymptomatic (WHO clinical stage I), respectively. This may be due to the fact that those HIV-positive patients with advanced immunosuppression may suffer from variety of opportunistic infections (OIs) and this suffering may lead to suicidal attempt.

HIV-positive patients who were not on HAART were 3.5 times more likely to have suicidal attempt as compared to those who were on HAART. The possible reason may be the fact that those HIV-positive patients who were not on HAART may think next of being on HAART and the probable outcome of lifelong drug side effect. Another possible reason may be patients thinking about future burden of lifelong treatment.

Participants with depression were two times more likely to have a suicidal attempt as compared to those with no depression. This is in agreement with other similar studies conducted in Uganda, New York, and Virginia University [11, 30]. This may be due to the fact that depressed individuals have feelings of hopelessness, guilt, and worthlessness and these may force them to attempt suicide [3].

The result of the present study revealed that ever use of the substance was another factor for suicidal attempt among HIV-positive participants. Those who ever used substance in their life were 3.4 times more likely to attempt suicide as compared to those who had no history of substance use in their life. This may be due to the fact that use of substance could disturb normal function of the brain, which could contribute to attempting suicide [11, 24, 30]. Another explanation may be the economic impact of substance use; that is, when people are substance abused, they may think of suicide if they are unable to buy it.

## 5. Limitation of the Study

In this study, we assessed suicidal ideation; individuals may not disclose their actual thought about suicide through an interview. Those comorbid medical and psychiatric problems that may be a factor for suicidal ideation and attempt were not assessed.

## 6. Conclusion

The prevalence of suicidal ideation and suicidal attempt was found to be high among HIV-positive patients. Advanced WHO clinical stage, not being on HAART, family history of the suicidal attempt, depression, and perceived stigma were associated with suicidal ideation and attempt.

## 7. Recommendation

Early diagnosis and treatment of opportunistic infections and depression as well as timely provision of ART need to be encouraged in HIV-positive adults. Furthermore, counseling on substance use and its consequences and early identification of HIV-positive people with family history of suicidal ideation have to be considered. Psychiatric evaluation needs to be done for those HIV-positive people using substances.

## Figures and Tables

**Table 1:** Sociodemographic characteristics of HIV-positive people at Zewditu Memorial Hospital, Addis Ababa, Ethiopia, June 2014 (*n* = 417).

Variables	Categories	Frequency (%)
Age in years	18–27	22 (5.3)
	28–37	139 (33.3)
	38–47	160 (38.4)
	48–57	75 (18.0)
	≥58	21 (5.0)

Sex	Male	174 (41.7)
	Female	243 (58.3)

Ethnicity	Amhara	231 (55.4)
	Oromo	63 (15.1)
	Tigre	75 (18.0)
	Gurage	37 (8.9)
	Other^*∗*^	11 (2.6)

Marital status	Married	188 (45.1)
	Single	116 (27.8)
	Divorced	57 (13.7)
	Widowed	56 (13.4)

Religion	Orthodox	333 (79.8)
	Muslim	32 (7.7)
	Protestant	42 (10.1)
	Catholic	10 (2.4)

Educational status	No formal education	9 (2.2)
	Primary	106 (25.4)
	Secondary	152 (36.5)
	Tertiary	150 (35.9)

Occupation	Unemployed	203 (48.7)
	Employed	214 (51.3)

Monthly income	<749	93 (22.3)
	750–1199	52 (12.5)
	≥1200	272 (65.2)

With whom you are living	Alone	138 (33.1)
	Partner	183 (43.9)
	Family	96 (23.0)

Social support	Good	205 (49.2)
	Poor	212 (50.8)

Depression	Presence	197 (47.2)
	Absence	220 (52.8)

Perceived stigma	Yes	234 (56.1)
	No	183 (43.9)

Ever substance use	Yes	68 (16.3)
	No	349 (83.7)

Current substance use	Yes	7 (1.7)
	No	410 (98.3)

Other^*∗*^ = Wolayta, Hadiya, and Afar.

**Table 2:** Logistic regression analysis of factors associated with suicidal ideation among HIV-positive people at Zewditu Memorial Hospital, Addis Ababa, Ethiopia, June 2014.

Variable	Categories	Suicidal ideation		COR (CI: 95%)	AOR (CI: 95%)
		Yes	No		
Sex	Male	28	146	1.00	1.00
	Female	66	177	1.94 (1.18–3.18)^*∗∗*^	1.67 (0.96–2.88)

Occupation	Unemployed	56	147	1.76 (1.10–2.81)	1.59 (0.94–2.68)
	Employed	38	176	1.00	1.00

Living with	Alone	38	100	1.90 (0.98–3.65)	1.53 (0.74–3.14)
	Partner	40	143	1.39 (0.73–2.65)	2.01 (0.98–4.08)
	Family	16	80	1.00	1.00

Social support	Good	36	169	1.00	1.00
	Poor	58	154	1.77 (1.10–2.82)^*∗∗*^	1.07 (0.56–2.01)

WHO clinical stages	I	26	155	1.00	1.00
	II	29	112	1.54 (0.86–2.76)	1.47 (0.77–2.79)
	III	28	45	3.71 (1.97–6.95)^*∗∗∗*^	4.12 (2.07–8.16)^*∗∗∗*^
	IV	11	11	5.96 (2.34–15.15)^*∗∗∗*^	6.55 (2.35–18.2)^*∗∗∗*^

Being on ART	Yes	82	297	1.00	1.00
	No	12	26	1.67 (0.80–3.45)	2.49 (1.07–5.70)^*∗*^

Family history of suicidal attempt	Yes	15	21	2.73 (1.34–5.53)^*∗∗*^	2.25 (1.01–5.03)^*∗*^
	No	79	302	1.00	1.00

Depression	Presence	62	135	2.69 (1.66–4.36)^*∗∗∗*^	2.45 (1.45–4.12)^*∗∗*^
	Absence	32	188	1.00	1.00

Social stigma	Yes	67	167	2.32 (1.41–3.81)^*∗∗*^	1.76 (1.02–3.03)^*∗*^
	No	27	156	1.00	1.00

^*∗*^ *p* value < 0.05, ^*∗∗*^*p* value < 0.01, and ^*∗∗∗*^*p* value < 0.001.

Model: Chi-square = 9.94, df = 8, and Sig. = 0.269.

**Table 3:** Logistic regression analysis of factors associated with suicidal attempt among HIV-positive people at Zewditu Memorial Hospital, Addis Ababa, Ethiopia, June 2014.

Variable	Categories	Suicidal attempt		COR (CI: 95%)	AOR (CI: 95%)
		Yes	No		
Sex	Male	13	161	1.00	1.00
	Female	45	198	2.82 (1.46–5.39)^*∗*^	4.48 (1.85–10.81)^*∗∗*^

Occupation	Unemployed	37	166	2.04 (1.15–3.63)^*∗*^	1.91 (1.001–3.65)^*∗*^
	Employed	21	193	1.00	1.00

Monthly income (Ethiopian Birr)	<750	20	73	2.21 (1.18–4.12)^*∗*^	1.58 (0.72–3.44)
	750–1200	8	44	1.47 (0.63–3.40)	1.06 (0.38–2.92)
	≥1200	30	242	1.00	1.00

Social support	Good	20	185	1.00	1.00
	Poor	38	174	2.02 (1.13–3.60)^*∗*^	1.63 (0.84–3.13)

WHO clinical stages	I	14	167	1.00	1.00
	II	16	125	1.53 (0.71–3.24)	1.47 (0.64–3.33)
	III	18	55	3.90 (1.82–8.36)^*∗∗∗*^	4.46 (1.93–10.29)^*∗∗∗*^
	IV	10	12	9.94 (3.65–27.04)^*∗∗∗*^	10.98 (3.56–33.79)^*∗∗∗*^

Started HAART	Yes	49	330	1.00	1.00
	No	9	29	2.09 (0.09–4.67)^*∗*^	3.44 (1.33–8.89)^*∗*^

Family history of suicidal attempt	Yes	10	26	2.67 (1.21–5.87)^*∗*^	2.26 (0.88–5.75)
	No	48	333	1.00	

Depression	Presence	38	159	2.39 (1.33–4.26)^*∗*^	2.04 (1.07–3.87)^*∗*^
	Absence	20	200	1.00	1.00

Perceived stigma	Yes	42	192	2.28 (1.23–4.21)^*∗∗*^	1.34 (0.60–3.01)
	No	16	167	1.00	1.00

Ever use of substance	Yes	13	55	1.59 (0.80–3.15)	3.39 (1.32–8.73)^*∗*^
	No	45	304	1.00	1.00

^*∗*^ *p* value < 0.05, ^*∗∗*^*p* value < 0.01, and ^*∗∗∗*^*p* value < 0.001.

Model: Chi-square = 11.11, df = 8, and Sig. = 0.19.
